# Autism, evolution, and the inadequacy of ‘spectrum’

**DOI:** 10.1093/emph/eoy025

**Published:** 2018-09-24

**Authors:** Neil S Greenspan

**Affiliations:** Department of Pathology, Case Western Reserve University, Cleveland, OH, USA

**Keywords:** phenotype, dimension, spectrum, genetic variant, fitness

## Abstract

Lay Summary: Individuals diagnosed with autism display variation in many traits, such as interest and ability in social interaction or resistance to change. Referring to this variation as a ‘spectrum’, defined as a range of values along an axis, understates the extent of such variation and can foster incorrect inferences.

In psychiatry, the currently accepted term for a developmental disability characterized by variably impaired social and communicative skills, repetitive behaviors, and restricted interests is “autism spectrum disorder.” “Spectrum,” typically refers to values of a variable distributed along a single dimension, incorrectly suggesting people with autism can be simply ranked as more or less ‘autistic.’ In fact, there are multiple traits that pertain to autism and that can vary somewhat independently, in part due to the evolutionary mechanisms that give rise to risk alleles. Therefore, a new and more accurate clinical descriptor should be adopted. I propose: autism-related disorders (ARD).

## THE LIMITATIONS OF ‘SPECTRUM’

The ‘Diagnostic and Statistical Manual of Mental Disorders, Fifth Edition’, which is the latest version of this widely used compendium of psychiatric conditions, employs the term ‘autism spectrum disorder’ or ‘ASD’ to refer to a range of conditions [1]. In the previous version (DSM-IV), the conditions included under the label ASD had been identified with the following terms [2]:

Autistic Disorder

Pervasive Developmental Disorder-Not Otherwise Specified (PDD-NOS)

Asperger Syndrome

Childhood Disintegrative Disorder (CDD)

Rett’s Disorder

‘ASD’ is intended to suggest that individuals diagnosed with an autism-related condition display core deficits in social communication and breadth of interests along with highly variable abnormal phenotypes relating to affective and behavioral traits, cognitive abilities, language skills and capacities, and other etiological factors and clinical manifestations. A recently published study, for example, makes use of nine dimensions of the phenotype for males and females with autism [3]. Even for the core or defining features there is substantial variation.

Unfortunately, the term ‘spectrum’, which is also sometimes applied to characterize other psychiatric conditions, such as schizophrenia and bipolar disorder [4, 5], is poorly suited to conveying variation in multiple variables, such as those cited in the preceding paragraph. There is value in exploring the reasons why this widely used term is not satisfactory and in recognizing the evolutionary genetic factors that account for the motivation to adopt a more appropriate word or phrase to encompass the family of conditions that can reasonably be classified together as ‘autism’.

The definitions of ‘spectrum’ offered by the online [6] Merriam-Webster dictionary (e.g. a continuous sequence or range) indicate that the word refers to a continuum along some axis. Other online dictionary definitions similarly support this interpretation. In other words, a spectrum refers to a collection of entities that can be ordered by reference to a single measurement constituting a 1D distribution. Herein lies the implicit conceptual difficulty with describing autism-related disorders (ARD) or the affected individuals as corresponding to a spectrum. ARD and the individuals affected of autism vary in multiple dimensions of relevance to diagnosis, clinical management, prognosis, research and qualification for government or charity-based services. These disorders or the people they affect cannot sensibly be ranked unambiguously as more or less ‘autistic’, a term accepted or rejected by different subsets of people diagnosed with ARD.

Of course, there are uses outside of the context of autism where the term ‘spectrum’ is employed for entities that cannot be reliably ordered along a single axis. That others use the term loosely does not strike me as a strong argument for being similarly unrigorous in discussing a group of disorders, especially when such usage has the potential for fostering serious and potentially consequential misunderstandings.

## DIVERSITY OF PHENOTYPES ASSOCIATED WITH AUTISM

**Figure 1.**
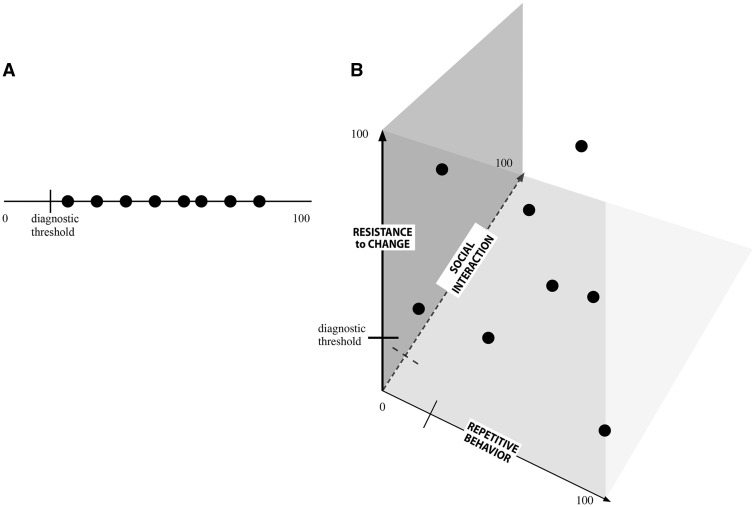
Graphical representations of two ways to conceptualize phenotypic variation among individuals affected by autism. (**A**) A single dimension of variation in the manifestation of symptoms associated with autism corresponding to a spectrum. (**B**) Illustrative plot showing a hypothetical 3D distribution of individuals exhibiting various degrees of autism-associated symptoms (as represented by repetitive behavior, social interaction and resistance to change). In both panels, each black dot represents a unique hypothetical individual with autism as defined by measurement of the relevant trait or traits

Assuming for the sake of discussion that all of the relevant variables can be assessed quantitatively with a reasonable degree of reproducibility, another way to frame the preceding point contrasts entities or attributes that can be usefully represented by one number versus those that require ordered sets of numbers (Fig. 1). For individuals with autism, the latter seems a much better fit, even if some of the variables that can be separately measured exhibit some degree of correlation. Nevertheless, these different attributes can occur independently of one another and are not so tightly correlated that it is unreasonable to assess them independently [7]. The variables used to label the three axes in Fig. 1B are used as examples to illustrate the key point that collapsing variation in three (or more) dimensions into variation in one dimension will necessarily lead to a loss of information. How particular genetic variants relate to the traits associated with autism can probably only be determined by studies that carefully document genotypes, phenotypes and those environmental factors that can be specified and measured or otherwise assessed.

Dr Lorna Wing, an English psychiatrist who had a daughter with autism, is often credited [8] with initiating use of the current terminology of ‘autism spectrum disorder’. She wanted to convey the extensive variability in patients diagnosed with autism.

Dr Wing made the following comments in the course of an interview with The Guardian in May of 2011 [8].

We should keep the descriptions of different groups but be less rigid. We need to see each child as an individual “to help them we need to understand all their particular skills, difficulties, behaviors and emotions”.

I interpret these statements to support a perspective on autism that explicitly acknowledges the multiple dimensions relevant to clinical descriptions of individuals with autism. These could include, but are not limited to, ability to relate socially, insight into social norms or recognition of social boundaries (which may not necessarily be closely correlated with interest in social contact), intellectual capabilities, frequency and fluidity of speech, tendency to become agitated, sensory sensitivities and the extent to which interests are restricted. Other clinically relevant variables that may or may not be directly related to the etiological factors causing the core symptoms of autism might include anxiety, obsessive-compulsive behavior and excessive vocalization.

The case against ‘spectrum’ is not, however, limited solely to its inadequacy for describing the variation in the population of individuals who are affected and clinically diagnosed. This term also has the potential to foster misguided and potentially damaging ideas about the family members in affected pedigrees and the trait variation in individuals more generally for which it might or might not make sense to apply the label ‘autism’.

## EVOLUTIONARY CONSIDERATIONS

It stands to reason that a process as complex as human neural development involves multiple pathways, signaling and metabolic, in multiple cell types [9]. Furthermore, given the centrality of reasoning and social cognition in human lives, it is highly likely that there has been strong selection favoring the effective integration of the many biological pathways that undergird these abilities [10]. Therefore, it might be expected that autism, like other conditions associated with altered neural development or development of other major physiological systems would arise to a significant degree through uncommon germline or *de novo* variants. This expectation is largely consistent with available data [11]. The large number of distinct loci involved and the existence of multiple autism-associated mutations at some of these loci likely contributes to the broad range of phenotypes encountered.

It is noteworthy that most of the genetic variants of large effect that are associated with autism are apparently causative in a single copy (i.e. the heterozygous state) [11]. Since autism has been shown to be associated with reduced evolutionary fitness not only of affected individuals but also of parents and male siblings [12, 13], it is considerably less likely that this cluster of conditions primarily results from genes positively selected in unaffected individuals, as posited by Ploeger and Galis [14].

However, it cannot currently be ruled out that common variants that modestly increase risk for autism are positively selected in other genetic backgrounds or were selected for in past environments.

While some of the autism-associated DNA sequences have been found to arise through *de novo* mutations in maternal or paternal germ cells or as somatic mutations in the neural stem cells of affected progeny, as noted above, others have originated in ancestors of the transmitting parent. In these latter instances, the sequences of concern were likely expressed in parental somatic cells. While the phenotypes attributable to these genes of at least grandparental origin may share similarities with those in the affected child, they may not be as extreme. In fact, the parental phenotypes may be sufficiently modest or within the range of normal to preclude justifying use of ‘autism’ with respect to the parent. For example, deletion of chromosome 1q21.1 can be associated with intellectual disability and, in some cases congenital abnormalities such as cardiac defects, and yet a parent from which the deletion was inherited may be affected only mildly or not at all [15]. In the context of schizophrenia, van Os makes similar arguments [16].

## UNFORTUNATE IMPLICATIONS OF ‘SPECTRUM’

While it should not be surprising that an affected child would resemble the parents in various traits to varying degrees, a diagnosis of autism in a parent or other family member should require convincing evidence of some significant level of dysfunction or disability, not merely an overlap in a subset of traits. A large fraction of individuals in the general population possess one or more traits, expressed to varying degrees, also possessed by individuals with autism.

Current terminology for the conditions collected together under the ‘ASD’ label has the potential to foster the mistaken belief that possessing a trait associated with autism, to any extent, makes one autistic, or as the unfortunate colloquialism has it, ‘on the spectrum’. This way of thinking drains the term ‘autism’ of meaning as a clinical diagnosis frequently associated with significant disabilities and challenges with great impact in daily life. As Lorna Wing noted [8]: ‘… nature never draws a line without smudging it. You cannot separate into those ‘with’ and ‘without’ traits as they are so scattered.’

## CONCLUSION

Therefore, I suggest that preferable terms for referring to the conditions that can usefully be classified as autism or to the individuals affected by autism are needed. I propose as a candidate term: ARD. There may be other formulations that serve equally well or better to convey the biological complexity of autism that the currently used phrasing fails to do with sufficient clarity. Whatever new term might ultimately be adopted by the field, it should be less likely to foster misguided and overly simplistic thinking about the application of ‘autism’ to people who have autism-related conditions or people who display one or a few putatively autism-associated traits but to an extent and in a context that renders the label misleading.

As noted above, there are multiple reasons for developing and refining a taxonomy of medical conditions that deserve the label autism: (i) diagnosis, (ii) prognosis, (iii) treatment, (iv) research and (v) qualification for services provided by government or charitable agencies. The uncomfortable truth associated with this reality is that there may be no one classification scheme that is optimal for all of these purposes. My goal here is not to definitively resolve this vexing issue that probably applies to many other neuropsychiatric disorders and other medical conditions. It is to call attention to and promote further thinking about the need to use terminology and conceptual frameworks that better facilitate rigorous thinking about the conditions that are appropriately labeled as ‘autism’ and the people merely affected or truly afflicted by them.
